# β-Cyclodextrin Attenuates Perfluorooctanoic Acid Toxicity in the Zebrafish Embryo Model

**DOI:** 10.3390/toxics5040031

**Published:** 2017-11-04

**Authors:** Mary Jo Weiss-Errico, John P. Berry, Kevin E. O’Shea

**Affiliations:** Department of Chemistry and Biochemistry, Florida International University, 11200 SW Eighth Street, Miami, FL 33199, USA; mweis047@fiu.edu

**Keywords:** perfluorooctanoic acid, β-Cyclodextrin, zebrafish embryo, host–guest chemistry, toxicity

## Abstract

Perfluorooctanoic acid (PFOA) has been linked to negative health outcomes including cancer, thyroid disease, infertility, and developmental delays. β-Cyclodextrin (β-CD), a cyclic sugar, has been previously shown to form strong host–guest complexes with PFOA, and is proposed as a means of environmental remediation with respect to this widespread contaminant. In the present study, β-CD was directly examined with regards to possible attenuation of the toxicity of PFOA specifically employing the zebrafish (*Danio rerio*) embryo model. Zebrafish embryos were exposed to various concentrations of PFOA without β-CD, and with equimolar (1:1) and excess (2:1) molar ratios of β-CD to PFOA, and assessed for lethality and developmental toxicity through seven days post-fertilization (dpf). Rapid onset of lethality with limited morphological abnormalities was observed at relatively low concentrations of PFOA (LC_50_ ≈ 50 ppm), along with effects on morphometric and neurobehavioral parameters in surviving embryos. A highly significant difference (*p* < 0.0001) was observed between the 2:1 treatment, and both 1:1 and PFOA only treatments, with respect to lethal concentration and apparent neurobehavioral effects, suggesting an effectively reduced toxicity of the fully complexed PFOA. In contrast, however, neither β-CD treatment reduced developmental toxicity with respect to the morphometric endpoint (i.e., interocular distance). Whereas LC_50_ of PFOA alone did not change over 7 dpf, the 1:1 and 2:1 values decreased slightly over time, suggesting either delayed or alternative toxic effects on later developmental stages at presumptively lowered levels. This study, therefore, indicates β-CD may be an effective agent to reduce toxicity of and mitigate environmental health concerns associated with PFOA, but that further study is required to elucidate the mechanism of complexation as it relates to the attenuation of toxicity.

## 1. Introduction

**Figure 1:**
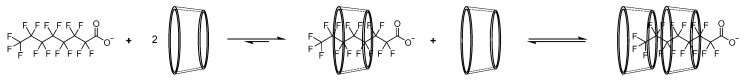
Host–guest complexes of β-cyclodextrin (β-CD) and perfluorooctanoic acid (PFOA), and equilibrium from free PFOA to the 1:1 β-CD:PFOA complex to the 2:1 β-CD:PFOA complex (from left to right). Cross-sectional areas of PFOA and β-CD, respectively, are 28.3 Å^2^ and 30.2 Å^2^ [18].

Perfluorooctanoic acid (PFOA) is a persistent organic pollutant and contaminant of emerging concern [1,2]. PFOA belongs to a large class of perfluorinated alkyl substances (PFASs). PFOA has been used historically in fluoropolymer synthesis, in aqueous film-forming foams, and in stain-, grease-, and water-repellants for consumer products [1,2,3,4]. The structure has seven perfluorinated carbons, and a carboxylic acid head group (Figure 1), making PFOA both hydrophobic and lipophobic. The numerous C-F bonds give the compound extreme chemical and thermal stability [1,2,5]. These unique properties cause PFOA to be both environmentally persistent and bioaccumulative [1,2,3,4,5,6,7,8]. Exposure specifically to PFOA has been linked to various cancers, including liver, kidney and bladder cancer, as well as thyroid disease, infertility, and developmental delays [5,6,7,8,9,10,11,12,13]. Although PFOA has been phased-out of production in the United States and Europe, it is still widely detected in the environment, wildlife, and humans [3,4,5,6,7,8,9]. A recent study found that over six million Americans have drinking water with PFOA concentrations above the U.S. Environmental Protection Agency’s lifetime advisory limit (70 parts per trillion) [4]. There is an urgent need to identify effective methods for environmental remediation, and other mitigation strategies (e.g., water treatment), with respect to the negative health effects of PFOA.

Cyclodextrins (CDs) have been proposed as a remediation strategy for a variety of pollutants, including perfluorinated surfactants such as PFOA [14,15,16,17,18,19,20,21,22]. Structurally, CDs are cyclic sugars made up of glucose monomers. They are inexpensive, water soluble, and non-toxic, and have been used not only in environmental remediation applications, but also as drug delivery systems for hydrophobic drugs [14,15,16,17]. β-Cyclodextrin (β-CD), made up of seven glucose monomers, has been shown to strongly encapsulate PFOA to form one of the strongest known CD-based host–guest complexes [18,19,20,21,22,23,24,25,26]. The fully fluorinated backbone of the PFOA alkyl chain interacts with the hydrophobic β-CD cavity via van der Waals interactions, while the PFOA carboxylate group can form hydrogen bonds with hydroxyl groups lining the cavity perimeter of β-CD (Figure 1). Furthermore, the β-CD cavity has an ideal cross-sectional area (30.2 Å^2^) to encapsulate PFOA (28.3 Å^2^) [18]. Both 1:1 and 2:1 β-CD:PFOA complexes (Figure 1) are formed in solution, with association constants of 5.0 × 10^5^ and 1.2 × 10^3^ M^−1^, respectively [18]. In a 1:1 ratio, the β-CD complexes at the middle of the PFOA backbone, whereas, at the 2:1 ratio, almost the full length of the PFOA chain is encapsulated by two adjacent β-CDs [18]. Since the encapsulation is strong, the presence of β-CD in PFOA-contaminated aqueous media dramatically reduces the concentration of uncomplexed PFOA, and, thus, is expected to inhibit its toxic effects.

To assess the potential of β-CD to attenuate the developmental and lethal effects of PFOA, we utilized the zebrafish (*Danio rerio*) embryo as a toxicological model. The zebrafish, and particularly embryonic and larval stages, have emerged as an important model system in a wide range of fields including assessment of chemical toxicity [27,28]. Practical advantages of the zebrafish embryo system include ease of rearing and a high fecundity, as well as small (~1 mm) and nearly transparent embryos, rapid (≤5–7 day) embryo development, and a fully sequenced genome. As such, the zebrafish embryo has been employed to look at a wide range of environmental contaminants in terms of acute and chronic toxicity [29,30,31]. Acute toxicological endpoints (e.g., embryotoxicity, teratogenicity, neurotoxicity), which are readily accessible in the zebrafish embryo model, are, furthermore, aligned with the reported health concerns associated with PFOA, and the system has, indeed, been used to evaluate toxicity of PFOA and related perfluorinated alkyl substances [32,33,34,35,36,37,38,39,40].

We propose that the β-CD:PFOA complex will effectively reduce the toxicity of PFOA in the zebrafish embryo. To test this hypothesis, relevant metrics of toxicity (e.g., lethal concentrations, developmental impairment, and neurobehavioral effects) were assessed for PFOA alone, and in the presence of equimolar (1:1) and excess (2:1) β-CD. The goal of the study was to evaluate potential reduction of toxicity in relation to complex formation. Although various human health effects have been linked to much lower environmental concentrations, relatively high concentrations of PFOA (i.e., parts-per-million (ppm) levels) were used in the current study rather than environmentally relevant concentrations such that acute toxicological endpoints, as a proxy of PFOA toxicity, could be assessed. This study, therefore, provides direct insight into the potential utility of cyclodextrins for attenuating the toxic effects of PFOA, and, thus, a key step toward future development of β-CD as a tool for mitigating the health effects associated with PFOA.

## 2. Materials and Methods

### 2.1. Test Chemicals

Perfluorooctanoic acid (PFOA, 96% purity) was purchased from Sigma-Aldrich (St. Louis, MO, USA). β-Cyclodextrin (β-CD, 98% purity) was purchased from Acros Organics (Geel, Belgium). Both chemicals were used without further purification. Stock solutions of PFOA (7.25 mM, i.e., 3000 parts-per-million (ppm)), 1:1 β-CD:PFOA (β-CD: 7.25 mM, PFOA: 7.25 mM), and 2:1 β-CD:PFOA (β-CD: 14.50 mM, PFOA: 7.25 mM) were prepared with deionized water in polypropylene tubes. The solutions were sonicated until dissolution of the solids was achieved. A control solution of deionized water was also stored in a polypropylene tube. Stock solutions were subsequently diluted over a relevant range of concentrations (see below) for assessment of zebrafish embryo toxicity.

### 2.2. Zebrafish Rearing and Breeding

To obtain embryos, zebrafish (PS strain) were reared and bred as previously described [41,42]. Briefly, adult zebrafish were maintained in 30-L tanks at 28 °C with 14 h:10 h light/dark cycle, and bred (from approximately 10–30 individuals) above 10-L tanks in mesh enclosures. Eggs were collected (from the bottom of tanks) within 1 h of the end of the dark cycle, and, following collection and washing, transferred to plates containing E3 medium [43]. Eggs containing dead, or obviously poor quality embryos, were removed. The remaining embryos were used, within ~2 h post-fertilization (hpf), for toxicity assays. All rearing and breeding was conducted under protocols approved by the University of Miami’s Institutional Animal Care and Use Committee (IACUC), and performed by trained investigators.

### 2.3. Zebrafish Embryo Toxicity Assay

Zebrafish embryo toxicity assays were adapted from previously described methods [41,42]. Assays were conducted in polypropylene 24-well plates (Evergreen Scientific, Los Angeles, CA, USA) with five embryos (4- to 32-cell stage) per replicate, i.e., well (*n* = 4), in E3 medium for a total of 20 zebrafish embryos per treatment/concentration [43]. Embryos were exposed via static exposure (i.e., without replenishment) to a range of PFOA concentrations (30, 50, 100, 150, 200, 250 and 300 ppm) alone, and in 1:1 and 2:1 ratios with β-CD, and subsequently observed at 1, 2, 3, 4 and 7 days post-fertilization (dpf) with a dissecting light microscope to assess mortality and relevant developmental toxicity. Exposures and assessments were repeated several times, in preliminary studies, to determine relevant concentration levels for PFOA, and generally confirm results. Lethality was calculated as the concentration corresponding to 50% mortality (LC_50_); the LC_50_ values, and their 95% confidence intervals, were calculated via Probit Analysis in SPSS (version 22.0; IBM Corporation, Armonk, NY, USA, 2013) [44]. In addition to lethality, inhibition of embryo development was morphometrically assessed based on the interocular distance between eyes (as a proxy for body size) of 7 dpf embryos (i.e., eleuthero-embryo stage) as measured using Olympus DP2-BSW imaging software (Olympus, Center Valley, PA, USA, 2009). Apparent neurobehavioral effects were additionally measured as the percent of 7-dpf eleuthero-embryos displaying listing (i.e., falling to one side) behavior within a 30 s period (with shaking between each measurement to allow embryos to right themselves). All toxicity assays involving zebrafish were conducted under protocols approved by the Florida International University’s Institutional Animal Care and Use Committee (IACUC), and performed by trained investigators.

### 2.4. Statistical Analyses

One-way analysis of variance (ANOVA) of the LC_50_ values, as well as the interocular distance and percent listing at 7 dpf, was performed in GraphPad Prism (version 5.03; GraphPad Software, Inc., La Jolla, CA, USA, 2010.) [45]. The significance level was set at *p* = 0.05.

## 3. Results

### 3.1. PFOA Toxicity in the Zebrafish Embryo Model

**Figure 2:**
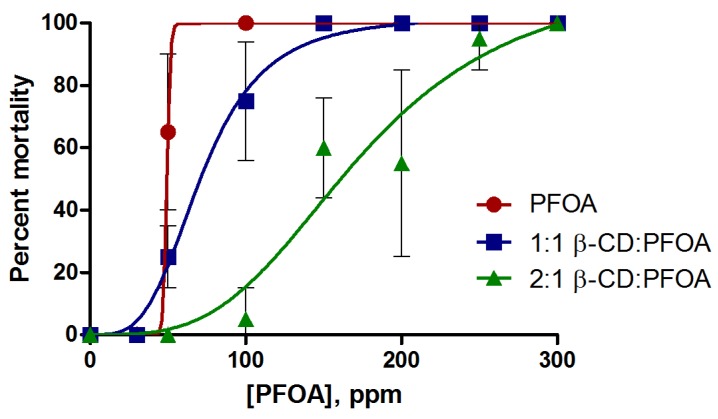
Concentration dependent toxicity of PFOA, and 1:1 and 2:1 β-CD: PFOA treatments, in terms of rapid onset lethality. Shown is percent mortality at 24 h post-fertilization (hpf) (error bars represent ± one standard deviation, *n* = 4).

**Figure 3:**
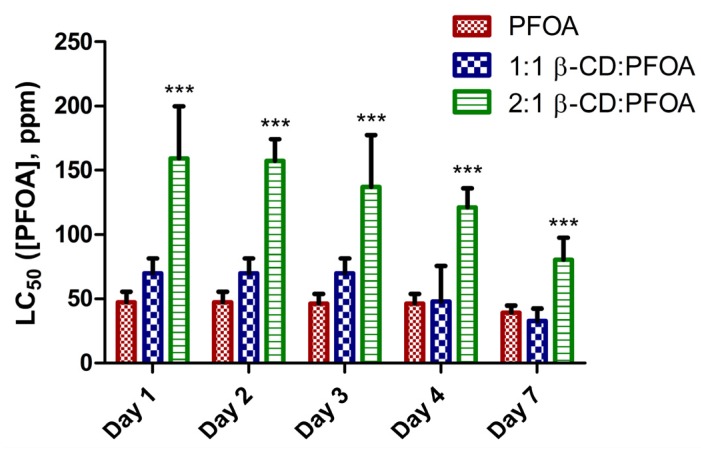
Calculated 50% lethal concentration (LC_50_) values for PFOA only, 1:1 β-CD:PFOA, and 2:1 β-CD:PFOA over 7 days post-fertilization (dpf). The 2:1 β-CD:PFOA LC_50_ values are significantly different (*** = *p* < 0.0001) than PFOA only, and 1:1 β-CD:PFOA. The LC_50_ values for PFOA only and 1:1 β-CD:PFOA are not significantly different from each other. Error bars represent 95% confidence intervals.

A rapid onset of embryotoxicity was observed for PFOA with mortality, characterized by coagulation of embryos, occurring within a few hours of exposure. By 24 hpf, a significant dose-dependent response with respect to lethality was observed (Figure 2) with all embryos dead at PFOA concentrations above 100 ppm. The observed dose response, with respect to mortality, remained largely unchanged, and the calculated LC_50_ values did not significantly change over the course of the exposure (i.e., 7 dpf; Figure 3). Notably, aside from a higher number of mortalities, no clear pattern of developmental deformities was observed: by 7 dpf, for example, only ~5% of both control and PFOA-treated embryos showed any discernible deformities, which included bent spines and edemas. Similarly, the hatching rates were unaffected with the majority of surviving embryos, in both control and PFOA treatments, hatched by 3 dpf.

**Figure 4:**
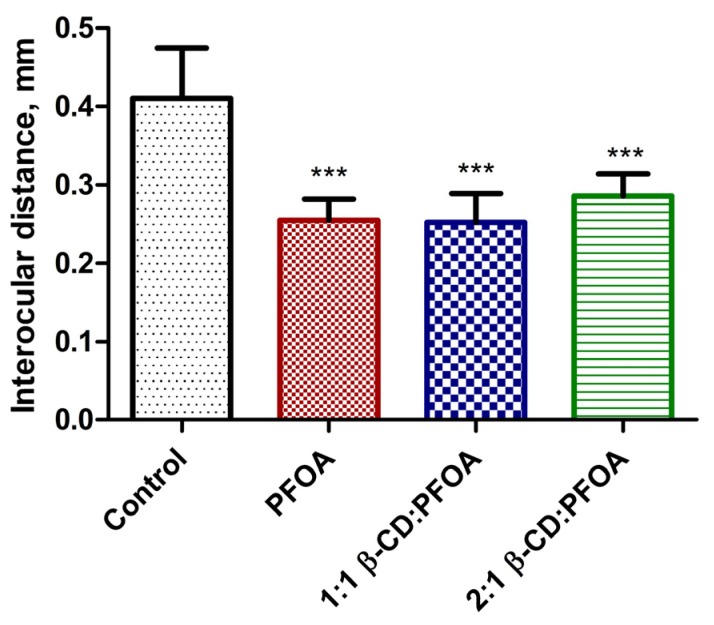
Interocular distance, as a morphometric measure of embryo body size, for untreated embryos (“Control”) compared to surviving embryos in sub-lethal concentrations (≤50 ppm) of PFOA only, and 1:1 and 2:1 β-CD:PFOA treatments, at 7 dpf. All three treatments are significantly different (*** = *p* < 0.0001) than controls. The three treatments are not significantly different from each other. Error bars represent 95% confidence intervals.

**Figure 5:**
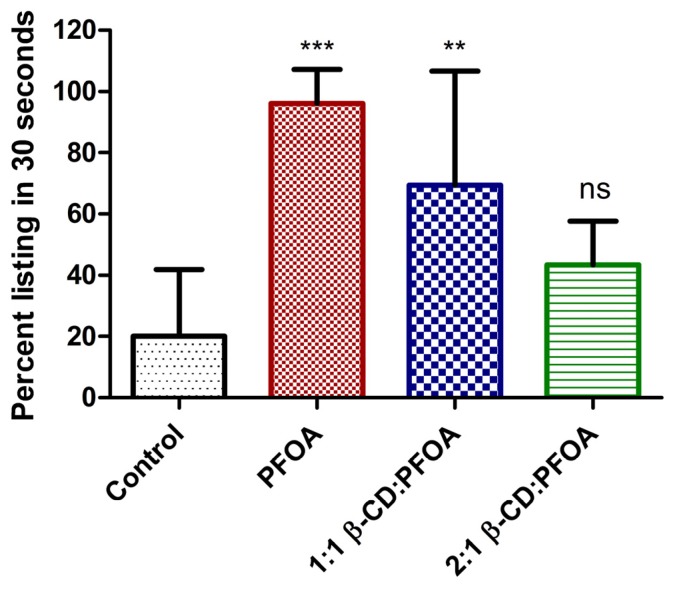
Percent listing after 30 s for the untreated embryos (“Control”) compared to surviving embryos in sub-lethal concentrations (≤50 ppm) of PFOA only, and 1:1 and 2:1 β-CD:PFOA treatments, at 7 dpf. The control values are significantly different from PFOA only (*** = *p* < 0.0001) and 1:1 β-CD:PFOA (** = *p* < 0.01). The 2:1 β-CD:PFOA values were not significantly different from controls (“ns”). Error bars represent 95% confidence intervals.

Apparent inhibition of embryo growth (i.e., reduced body size), however, was observed, with the surviving PFOA-treated embryos being smaller at the end of the exposure (i.e., 7 dpf) compared to controls. Inhibition of development was specifically evaluated morphometrically based on interocular distance at 7 dpf: significantly (*p* < 0.0001) reduced interocular distances were measured for surviving PFOA-treated embryos at sub-lethal concentrations (i.e., ≤50 ppm, pooled; 0.25 ± 0.06 mm) versus untreated control (0.41 ± 0.14 mm) embryos (Figure 4). In terms of other relevant endpoints, apparent neurobehavioral effects were observed for surviving embryos, and specifically a high frequency of listing was observed for PFOA-treated embryos. At low PFOA concentrations below the LC_50_ (i.e., ≤50 ppm, pooled), the percent of surviving embryos observed to list within a 30 s period (96 ± 9%) was significantly higher (*p* < 0.0001) than controls without PFOA (20 ± 13%; Figure 5).

### 3.2. Attenuation of PFOA Toxicity by β-CD

To evaluate the ability of β-CD to reduce the toxicity of PFOA, the LC_50_ values for each treatment (PFOA alone, 1:1 and 2:1 β-CD:PFOA ratios) were calculated and compared. At 1 dpf, a significantly (ANOVA, *p* < 0.0001) higher LC_50_ was observed for 2:1 β-CD:PFOA (159.3 ± 22.9 ppm) compared to both PFOA alone (47.3 ± 3.6 ppm), and the 1:1 β-CD:PFOA treatment (69.9 ± 5.7 ppm). The lethal concentration for 1:1 β-CD:PFOA was higher than PFOA alone, but the difference was not statistically significant. However, whereas LC_50_ did not change over time for PFOA alone, calculated values for both 1:1 and 2:1 β-CD:PFOA treatments notably decreased over 7 days of exposure (Figure 3) due to additional, post-hatch mortalities. By 4 dpf, the 1:1 β-CD:PFOA LC_50_ (48.1 ± 14.2 ppm) was essentially equal to the PFOA alone. Although LC_50_ for 2:1 β-CD:PFOA treatment decreased (e.g., 80.5 ± 9.3 ppm by 7 dpf), it was still significantly (*p* < 0.0001) higher than either PFOA alone, or PFOA in a 1:1 ratio with β-CD. No discernible toxicity was observed for β-CD alone, within the range of tested concentrations, including the maximum concentration (i.e., 1645 ppm, or 1.45 mM, β-CD) evaluated in the 2:1 ratio treatments.

In contrast to lethality, β-CD did not reduce apparent developmental toxicity in terms of a morphometric variable, i.e., interocular distance (Figure 4). Comparing surviving embryos at, or below, the LC_50_ (i.e., ≤50 ppm), the measure of interocular distance for 7 dpf embryos was significantly (*p* < 0.0001) lower in both 1:1 β-CD:PFOA (0.25 ± 0.07 mm) and 2:1 β-CD:PFOA (0.29 ± 0.07 mm) treatments when compared to controls, and nearly identical to PFOA alone (see above). Similar to lethality, on the other hand, the apparent neurobehavioral effect (i.e., listing) among 7 dpf embryos was reduced among surviving embryos at sub-lethal concentrations (i.e., ≤50 ppm) in the 2:1 β-CD:PFOA treatment: percent listing in this treatment was not significantly different from untreated controls (Figure 5). Frequency of listing in the 1:1 treatment was decreased, compared to that of PFOA alone, but it was still significantly higher (*p* < 0.01) than untreated controls.

## 4. Discussion

Toxicity of PFOA in the zebrafish embryo model, including embryotoxicity (i.e., lethality), reduced body size, and neurobehavioral effects observed in the present study, is generally consistent with prior studies [32,33,35]. Aligned with these previous studies [33], a rapid onset of lethality (≤24 hpf), and relatively limited occurrence of developmental deformities, including bent spines and edemas were similarly observed in the current study. Lethal concentrations for PFOA (i.e., LC_50_ ≈ 50 ppm; Figure 3) in the current study, however, were considerably lower than measured in prior studies, which have typically reported LC_50_ values above 500 ppm and/or test ranges [32,40], although others [33,35] have reported somewhat lower values (e.g., 262 and 371 ppm, respectively). It has been suggested [37] that the LC_50_ of PFOA may be, in fact, ten times lower than originally reported, which would put it within the range presently observed. Variability in lethal concentrations is presumably due to genetic differences of zebrafish lines used and/or other experimental conditions, and were not addressed further in the present study. Both reduced body size (Figure 4) and locomotory effects (Figure 5) have been, likewise, reported for PFOA-treated zebrafish embryos at sub-lethal concentrations [32,37,38,39]. Notably, these sub-lethal effects were observed in previous studies [38,39] at considerably lower (i.e., micro- to nanomolar) exposure concentrations, and, interestingly, effects extended into (following embryonic exposure) adult stages.

Consistent with formation of host–guest complexes, β-CD generally reduced toxicity of PFOA (Figure 3 and Figure 5). Although a 1:1 ratio decreased both rapid lethality at early embryonic stages (i.e., ≤3 dpf), and neurobehavioral effects, only the measured decreases (approximately two-fold) associated with the 2:1 ratio were statistically significant through 7 dpf (Figure 3). That being said, neither β-CD treatment significantly attenuated a morphometric variable (interocular distance) indicative of reduced embryo growth and body size associated with PFOA toxicity (Figure 4). Furthermore, while the LC_50_ of PFOA alone did not change during the exposure, values decreased over 7 dpf for both 1:1 and 2:1 ratios (Figure 3). These latter observations suggest a toxicity toward post-hatch larvae at either presumably lowered concentrations of non-complexed PFOA or alternatively less toxic and/or bioavailable complexes, which are uncoupled from the potent embryotoxicity associated with rapid onset lethality at early embryonic stages.

The mechanism whereby β-CD reduces toxicity via complex formation remains to be clarified in future studies. However, the current state of knowledge with respect to the formation of the β-CD:PFOA host–guest complex points to a few possibilities. It appears that the ratio of β-CD, rather than the concentration of β-CD, determines the inhibition of PFOA toxicity. Previous studies have shown that, at equimolar concentrations, PFOA is strongly complexed by β-CD with a 1:1 β-CD:PFOA association constant of 5.0 × 10^5^ M^−1^, and, furthermore, suggest that the complex is not significantly disturbed under different water quality conditions (e.g., ionic strength, pH, presence of humic acid, and presence of competing model pollutants) [18]. In addition, a 2:1 complex can form (Figure 1), particularly in the presence of excess β-CD (as expected in the 2:1 ratio treatment). Differences in bioavailability, and thus uptake, of the two complexes, may explain the differential toxicity observed between 1:1 and 2:1 β-CD treatments. Indeed, it has been shown that relative lipophilicity significantly effects and is correlated with uptake in the zebrafish embryo model [46]. It would be expected that increased complexation of PFOA would decrease log P values and, thus, uptake.

This model (Figure 1), however, largely assumes stoichiometric, stepwise and stable host–guest complexes. Alternatively, it is possible that non-stoichiometric and/or non-sequential complex formation could lead to, likewise, non-stoichiometrically reduced levels of free PFOA which would, in turn, be quantitatively dependent on the relative β-CD concentration. Such an alternative model could, therefore, explain quantitative differences in toxicity between 1:1 and 2:1 ratios whereby, for example, formation of “2:1 complexes” under equimolar (1:1) conditions would lead to persistence of uncomplexed (and thus toxic) PFOA. Presence of free PFOA in solution, and corresponding sub-lethal toxicity might, in this regard, be further exacerbated by bioaccumulation of PFOA, and subsequent release at later stages. Indeed, previous studies have shown high levels of bioconcentration of PFOA in zebrafish embryos including intestine and bile (and potential enterohepatic recirculation), as well as yolk, as a means of bioaccumulation and release at later embryonic stages [36]. Competition between bioaccumulation and complex formation might, more generally, explain toxicity at higher concentrations even in 2:1 treatments including both post-hatch toxicity (Figure 3), and inability of either β-CD treatment to effectively reduce developmental toxicity, in terms of morphometric parameters (Figure 4). Quantitative analysis of PFOA and β-CD complexes with respect to both exposure medium and bioaccumulation/uptake (e.g., critical body residue), including tissue specific bioavailability, in future studies could address these interrelated mechanisms.

## 5. Conclusions

The results of this study directly suggest that β-CD, particularly in excess molar ratios, can largely attenuate the toxicity of PFOA in solution as evidenced by reduced toxicity in the zebrafish embryo model. The ratio of β-CD to PFOA, rather than the concentration of β-CD, drives this attenuation. It is proposed that the host–guest complex may exhibit less biological activity, or alter bioavailability, compared to PFOA alone. Alternatively, β-CD may simply reduce by way of the variable formation of 1:1 and 2:1 complexes levels of free (and thus toxic) PFOA in solution in a concentration-dependent manner. Further research is needed to understand the mechanism of decreased toxicity, as well as to determine efficacy of β-CD at environmentally relevant concentrations of PFOA, and with respect to non-acute toxicological endpoints (specifically relevant to human health). However, these findings suggest that β-CD is a potentially promising tool for environmental remediation, and mitigation of toxic effects (e.g., drinking water treatment), of PFOA and, perhaps, related perfluorinated compounds as they relate to environmental health concerns.
